# SARS-CoV-2 and interferon blockade

**DOI:** 10.1186/s10020-020-00231-w

**Published:** 2020-11-09

**Authors:** Betty Diamond, Bruce T. Volpe, Sonya VanPatten, Yousef Al Abed

**Affiliations:** 1 Center for Molecular Medicine, The Feinstein Institute for Medical Research, 350 Community Drive, Manhasset, NY 11030 USA; 2 Center for Bioelectronic Medicine, The Feinstein Institute for Medical Research, 350 Community Drive, Manhasset, NY 11030 USA

## Abstract

The response to viral infection generally includes an activation of the adaptive immune response to produce cytotoxic T cells and neutralizing antibodies. We propose that SARS-CoV-2 activates the innate immune system through the renin-angiotensin and kallikrein-bradykinin pathways, blocks interferon production and reduces an effective adaptive immune response. This model has therapeutic implications.

Patients suffering from Covid-19, the disease caused by SARS-CoV-2, have uncontrolled inflammation (Tay et al. 2020). Often in the context of infection, this is thought to represent cytokine storm with a major contribution from lymphoid cells. However, there is little evidence of excessive lymphoid activation in the blood of infected individuals; in fact, severe infection is characterized by lymphopenia and patients with severe infection exhibit a high neutrophil to lymphocyte ratio (Guan et al. 2020). Moreover, while there is an antibody response that develops in infected individuals, a detectable antibody response can be achieved without massive lymphoid activation (Quinti et al. 2020). Here we propose that the systemic inflammation seen in Covid-19 patients results from the activation of two intersecting systems, the renin-angiotensin system (RAS) and the kallikrein-bradykinin system (Diamond 2020). These two systems together can serve to promote inflammation without activating an adaptive immune response. Moreover, their activation diminishes production of type 1 interferon leading, we propose, to a pathologic condition in Covid-19 patients characterized by systemic inflammation and sustained viral replication.

Both the RAS and the kallikrein-bradykinin system have long been appreciated for their importance in vascular biology (Gobel et al. 2019). Both also contribute to immune modulation (Garvin et al. 2020; Seliga et al. 2018). Angiotensin II, a major effector molecular in the RAS, is derived from angiotensin I through the action of angiotensin converting enzyme (ACE) (Donoghue et al. 2000). Angiotensin II has 2 receptors, AT1 and AT2, that are expressed on a broad range of cells (Clarke et al. 2012). The binding of angiotensin II to AT1 promotes vasoconstriction but also promotes inflammation, with activation of NFκB dependent cytokines but not type 1 interferon (Benigni et al. 2010). Engagement of AT2 by angiotensin II, in contrast, induces vasodilatation and IL-10 production (Crowley and Rudemiller 2017). Under inflammatory conditions, AT1 expression is increased, thereby amplifying an inflammatory program (Crowley and Rudemiller 2017; Koka et al. 2008; Tikellis and Thomas 2012). Of importance to our understanding of Covid-19 pathology, angiotensin II can block monocyte to dendritic cell differentiation impairing the initiation of an adaptive immune response (Ingersoll et al. 2011) and can also cause T cell apoptosis (Odaka and Mizuochi 2000), thereby limiting the contribution of the adaptive immune response and contributing to the lymphopenia of Covid-19 patients.

ACE2 is a membrane-bound protease that cleaves angiotensin II to produce ang1-7, a peptide which can bind Mas, a G protein coupled receptor (Gheblawi et al. 2020). This receptor ligand interaction initiates vasodilatation and an anti-inflammatory program. Thus, angiotensin II can be either pro or anti-inflammatory depending on the relative expression of AT1, AT2 and ACE2 (Crowley and Rudemiller 2017; Koka et al. 2008; Tikellis and Thomas 2012). A major function of ACE2 is to reduce the amount of angiotensin II, in addition, angiotensin II and ACE2 often have contrasting effects. In particular, angiotensin II facilitates release of HMGB1 from numerous cell types and ACE2 inhibits its release (Zhou et al. 2018). HMGB1 is a pro-inflammatory cytokine or a chemokine depending on its redox state (Andersson and Tracey 2011). It is important in myeloid cell activation, but it also affects hematopoiesis, aborting erythropoiesis and skewing to myelopoiesis and away from lymphopoiesis (Valdes-Ferrer et al. 2015). We suggest this molecular pathway, therefore, may also contribute to the lymphopenia seen in Covid-19 patients.

ACE is important not only because it converts angiotensin I to angiotensin II, but also because it degrades bradykinin. Bradykinin arises through the kallikrein bradykinin pathway (Seliga et al. 2018). It has 2 receptors (Bhoola et al. 1992). BR2 is constitutively expressed on many cells (Marceau and Regoli 2004). The bradykinin-BR2 interaction leads to vasodilatation and suppresses type 1 interferon production (Seliga et al. 2018). BR1, which is induced during inflammation, is involved in amplifying inflammatory pathways. Thus, high ACE favors vasoconstriction and inflammatory cytokines by increasing available angiotensin II and decreasing available bradykinin. Low ACE decreases inflammatory cytokines and permits type 1 interferon production (Crowley and Rudemiller 2017; Koka et al. 2008; Tikellis and Thomas 2012; Hadjadj et al. 2020).

These pathways intersect with the SARS-CoV-2 virus, as ACE2 is the cellular receptor for the spike protein of the virus (Lan et al. 2020). When ACE2 is engaged by the virus, ADAM17 (also called TACE) is activated to cleave ACE2 from the membrane. Soluble ACE2 is less effective at converting pro-inflammatory angiotensin II into ang1-7 and biases the RAS toward inflammation (Simoes et al. 2013). The engagement of these pathways helps explain how severe Covid-19 infection is characterized by massive inflammation in multiple target organs, a poor anti-viral response with little production of interferon, and little participation of the adaptive immune system. Indeed, it is the interferon pathway that may be most important in conferring protection against severe disease as agammaglobulinemic individuals do not appear to be at increased vulnerability to infection with SARS-CoV-2 (Quinti et al. 2020).

There are three major cell types in this model that mediate acute severe Covid-19 symptomatology: myeloid cells, both neutrophils and macrophages, microglia, and endothelial cells. Macrophages and neutrophils in blood are clearly activated (Vabret et al. 2020). There was evidence of microglial activation in patients succumbing to SARS (Xu et al. 2005); the data on microglial activation in SARS-CoV-2 patients is not yet available. There is evidence for endothelial cell activation (Reichard et al. 2020) with reports of massive thrombosis in Covid-19 patients (Oxley et al. 2020). While the overall data on endothelial cell activation are indirect (Herman et al. 2020), the case reports are compelling (Mao et al. 2020; Solomon et al. 2020; Jaunmuktane et al. 2020; Ellul et al. 2020).

There are three major organs to consider in individuals with severe Covid-19 infection.

## Covid-19 and the lungs

Respiratory transmission appears to be the primary route of infection in adults as both nasal and lung epithelial cells express ACE2 (Tay et al. 2020). The lower infection rate in children may relate, in part, to the lower expression of ACE2 on their nasal epithelium (Patel and Verma 2020). Infection of alveolar epithelial cells by virus leads to their death through cytopathic effects of the virus, and consequently to hypoxia. Hypoxia upregulates HIF1α leading to impaired interferon production but intact production of proinflammatory cytokines (Wobben et al. 2013). In the lung, bradykinin causes fibroblasts to produce chemoattracts for neutrophils (Ehrenfeld et al. 2006). Both angiotensin II and bradykinin increase vascular permeability, leading to extravasation of neutrophils into the lung. As serum from SARS-CoV-2 patients has been shown to induce netosis (Wang et al. 2020), it is highly likely that netosis occurs within alveolae further compromising lung function and leading to acute respiratory distress syndrome in some (Vabret et al. 2020).

## Covid-19 and the brain

The effect of Covid-19 on the central nervous system has received some attention, but perhaps not enough. The virus responsible for SARS was found in brain tissue and the CSF (Xu et al. 2005; Hung et al. 2003). In general, virus may enter the brain by infecting leucocytes that penetrate a compromised blood brain barrier (BBB), by infecting brain-microvascular endothelium to enter the brain, or by direct penetration of nerves or microglia (Koyuncu et al. 2013). In the case of a virus like SARS-CoV-2 which is transmitted by inhalation, the virus most likely enters through olfactory bulb epithelial cells (Bilinska et al. 2020), and then enters ACE2 expressing microglia and neurons (Xu et al. 2005; Jaunmuktane et al. 2020; Ellul et al. 2020), although other recent studies suggest that non-neuronal cell types play a role in the development of anosmic symptoms (Bilinska et al. 2020; Brann et al. 2020). This direct entry of virus into brain parenchyma might account for the fact that anosmia is often a presenting symptom of Covid-19 infection. Once brain cells are infected and there is evidence for infection by glia by SAR-CoV-2, the RAS can be activated locally to initiate neuroinflammation. Microglia produce angiotensinogen, the precursor to angiotensin I and neurons produce ACE; both cell types express AT1 (Lanz et al. 2010). That the RAS can be important in sustaining neuroinflammation has been seen in Alzheimer’s disease and in a model of neuropsychiatric SLE (Oliveira et al. 2014; Nestor et al. 2018; Nocito et al. 2020). Moreover, HMGB1, which is released by activated microglia, potentiates the activation of the *N*-methyl d-aspartate receptor (NMDAR) on excitatory neurons (Balosso et al. 2014), with excessive activation leading to excitotoxic neuron death. The greater the activation of NMDARs, the greater release of proinflammatory HMGB1 by neurons, creating a positive feedback loop. Moreover, type 1 interferon induces production of quinolinic acid by microglia through an indolamine 2,3-dioxygenase (lDO) dependent pathway (Kwidzinski and Bechmann 2007); quinolinic acid is an NMDAR agonist. Thus, the amount of interferon that is induced by the SARS-CoV-2 virus may be inadequate for controlling viral replication but capable of enhancing an inflammatory environment that facilitates excitotoxic neuron death. In this model, viral infection in the brain can generate self-sustaining pathologic neuroinflammation. Indeed, as survivors of severe infection are followed through the recovery stage it is clear that many suffer from persistent cognitive impairment which may represent both neuronal loss and neuronal dysfunction (Helms et al. 2020).

## Covid-19 and the vasculature

Angiotensin II binds AT1 on endothelial cells and leads to release of HMGB1 (Zhou et al. 2018; Nair et al. 2015), chemokines and pro-inflammatory cytokines and to increased vascular permeability. Of critical importance, activation of AT1 on endothelial cells also leads to increased expression of tissue factor which is necessary for thrombosis (Kunieda et al. 2003), a well appreciated and common morbidity in individuals with Covid-19. The combination of high expression of tissue factor on activated endothelial cells and activated monocytes favors a procoagulation state. Many Covid-19 patients also produce anti-phospholipid antibodies which also contribute to thrombosis (Zhang et al. 2020a).

This model applies to those with severe infection, many of whom have preexisting conditions, such as obesity or being elderly, that lead to increased angiotensin II and AT1, or reduced ACE2 (Feraco et al. 2013; Hajifathalian et al. 2020; Chung et al. 2020). There appears, in contrast, to be a subset of Covid-19 patients who have mild symptoms and develop high antibody titers. We hypothesize that these individuals produce high levels of type 1 interferon which control the virus and deviate the B cell response to extrafollicular differentiation of plasma cells leading to high titers of antibody but no memory B cells. Indeed, survivors of SARS exhibited a lack of memory B cells (Wec et al. 2020). We would suggest that these individuals have a RAS skewed to higher ACE2 and anti-inflammatory pathways, and to pathways that do not inhibit production of type 1 interferon.

**Fig. 1:**
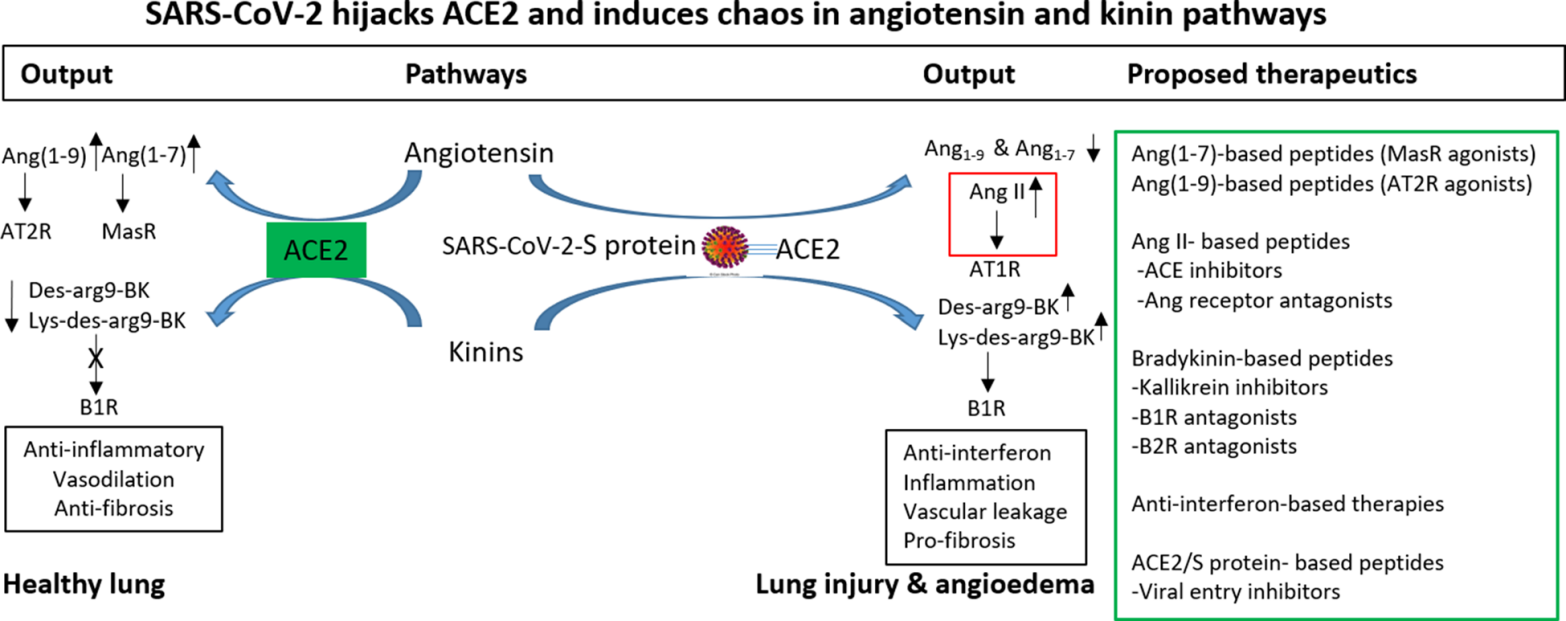
ACE2 is a key enzyme in the RAS, catalyzing the metabolism of Ang II to Ang(1-7) and Ang I to Ang(1-9). ACE2 also mediates degradation of ACE-catalyzed breakdown products, Des-arg9-Bk (B1R agonist) and Lys-des-arg9-Bk. The net result of ACE2 in these two systems is to counterbalance ACE/Ang II/AT1R and Bradykinin/Des-arg9-Bk/B1R pathways. Through its cellular binding and entry mechanisms, SARS-CoV-2 is proposed to result in a reduction of ACE2, leading to elevations in Ang I and II, and leading to AT1R stimulation, and Des-arg9-Bk leading to B1R stimulation thus exacerbating inflammation, vascular leakage, and pro-fibrotic events. Potential therapeutics include those targeted to angiotensin and bradykinin system related peptides, in addition to peptides targeting the ACE2-viral spike (S) protein interaction

Current and potential therapeutic interventions:

Interferon is likely the most important intervention at the beginning of the viral infection as it might block viral replication. It will be less effective in already severe infection characterized by inflammation (Sheahan et al. 2020; Mueller 2020). Here (Fig. 1), we emphasize the need to explore interventions that specifically target the RAS or kallikrein-bradykinin pathway which we hypothesize are the major contributors to severe inflammation.

Several candidate therapeutics focus on the virus, targeting viral replication (remdesivir), viral entry (Arbidol, APN01, convalescent plasma, monoclonal antibodies (REGN-COV2), camostat mesylate), or critical viral proteins (protease inhibitors). Therapies targeting other viral entry receptors including CD147 (Ulrich and Pillat 2020) and GPR178 (Ulrich and Pillat 2020; Elfiky 2020) are other avenues of investigation. Several promising therapeutics are designed to block viral entry pathways or prevent propagation and spread of the virus in susceptible organs. These may, like interferon, be useful early in disease. Other therapies focus on host inflammatory mediators (IFN-α, IFN-β, TZLS-501:IL-6 specific mAb (monoclonal antibodies), TJM2:GM-CSF-directed Ab). Some of these strategies and others are reviewed in (Tay et al. 2020; Tu et al. 2020; Lima et al. 2020). Most recently, the FDA approved remdesivir for emergency use authorization (EUA) in COVID-19. At the same time, the World Health Organization (WHO) discontinued the hydroxychloroquine and lopinovir/ritonavir arms of its Solidarity trial (https://www.who.int/news-room/detail/04-07-2020-who-discontinues-hydroxychloroquine-and-lopinavir-ritonavir-treatment-arms-for-COVID-19. The only successful therapy to date to dampen host inflammation is dexamethasone, which affects multiple pathways and cell types. There has been controversy regarding targeting the RAS. Initially, there was concern that ACE inhibitors might exacerbate disease, but studies suggest they may, in fact, be of modest benefit (Patel and Verma 2020; Zhang et al. 2020b). Angiotensin receptor antagonists (losartan and telmisartan; ClinTrials.gov: NCT04355936) also warrant consideration. Some therapies in development are now also addressing the RAS. As ACE2 is the main viral entry receptor, ACE2 receptor antagonists based on peptides from the viral spike protein are being synthesized and tested, in addition to spike protein-targeted antagonists derived from critical binding regions in the ACE2 receptor (VanPatten et al. 2020). Therapeutics which target TACE and would inhibit ACE2 shedding (Haga et al. 2010) have also received consideration.

As we have hypothesized that some of the inflammation induced in severe, and perhaps even moderate, Covid-19 is the result of dysregulation of the RAS and kallikrein-bradykinin pathways, the associated players serve as potential therapeutic targets (Fig. 1) As mentioned above, ACE inhibitors and AT1 blockers (ARBs) are approved and safe drugs. These pathways could be also be targeted with AT2 receptor-directed agonists (such as Angiotensin(1–9), Mas R agonists-AV0991, Angiotensin(1–7) (Zemlin et al. 2020; Paz Ocaranza et al. 2020). Bradykinin receptor antagonists are of potential interest, as bradykinin suppresses interferon production, enhances inflammation and causes vascular permeability (van de Veerdonk et al. 2020; Roche and Roche 2020). Therapeutics aimed to address the imbalance in angiotensin and kallikrein products pathway as well as inflammatory mediators such as HMGB1 are promising areas of future research in Covid-19 (Andersson et al. 2020). We propose that expedited development of therapeutics that target immune system modifiers focusing on those in the RAS and kallikrein/bradykinin pathway offer the most promising avenues for effective treatment of severe disease.

The rapid sharing of global research information and the publication of novel hypotheses regarding both pathogenesis and therapy have greatly enhanced our understanding of this virus, and the lessons learned from ongoing clinical trials will continue to guide future research and therapeutics development.
